# Assessment of Cimetropium Bromide Use for the Detection of Gastric Neoplasms During Esophagogastroduodenoscopy

**DOI:** 10.1001/jamanetworkopen.2022.3827

**Published:** 2022-03-23

**Authors:** Sang Yoon Kim, Jae Myung Park, Hyun Sun Cho, Yu Kyung Cho, Myung-Gyu Choi

**Affiliations:** 1 Department of Internal Medicine, Myoungji Hospital, Hanyang University College of Medicine, Goyang-si, Gyeonggi-do, Republic of Korea; 2 Graduate School, Department of Internal Medicine, College of Medicine, The Catholic University of Korea, Seoul, Republic of Korea; 3 Division of Gastroenterology and Hepatology, Department of Internal Medicine, College of Medicine, Seoul St. Mary’s Hospital, The Catholic University of Korea, Seoul, Republic of Korea; 4 Catholic Photomedicine Research Institute, Seoul, Republic of Korea; 5 Department of Health Promotion Medicine, Seoul St. Mary’s Hospital, The Catholic University of Korea, Seoul, Republic of Korea

## Abstract

**Importance:**

Esophagogastroduodenoscopy (EGD) is a common procedure used to examine upper gastrointestinal diseases. Although cimetropium bromide and other antispasmodic agents are commonly administered as premedication to inhibit peristalsis during EGD examination, there are few data regarding the benefits of cimetropium bromide for the detection of gastric neoplasms.

**Objective:**

To investigate the association between the use of cimetropium bromide as premedication and gastric neoplasm detection rates during EGD examination.

**Design, Setting, and Participants:**

This propensity score–matched retrospective cohort study included 67 683 participants who received EGD screening at the Health Promotion Center of Seoul St. Mary’s Hospital, The Catholic University of Korea, from January 2, 2010, to June 30, 2017. Data were analyzed from April 1 to December 30, 2021.

**Exposures:**

Participants were divided into 2 groups: those who received cimetropium bromide before EGD examination (intervention group) and those who did not (control group).

**Main Outcomes and Measures:**

Gastric neoplasm detection rates.

**Results:**

Among 67 683 participants, the mean (SD) age was 48.6 (10.8) years, and 36 517 participants (54.0%) were male; all participants were Asian (a racially homogenous population). Of those, 28 280 participants (41.8%; mean [SD] age, 50.3 [10.6] years; 57.8% male) received cimetropium bromide, and 39 403 participants (58.2%; mean [SD] age, 47.4 [10.8] years; 51.2% male) did not. Propensity score matching based on confounding variables yielded 41 670 matched participants (20 835 pairs). Detected lesions included 52 dysplasias (0.12%), 40 early cancers (0.10%), 7 advanced cancers (0.02%), and 3 lymphomas (0.01%). Gastric neoplasm detection rates were significantly higher in the intervention group (63 participants [0.30%]) vs the control group (39 participants [0.19%]; *P* = .02). A significant difference in the combined detection rate of dysplasia and early gastric cancer was found between those in the intervention group (57 participants [0.27%]) vs the control group (35 participants [0.17%]; *P* = .02). For small gastric lesions (<1 cm), those who received cimetropium bromide had higher detection rates (24 participants [0.12%]) than those who did not (11 participants [0.05%]; *P* = .03). Lesions in the gastric body were detected significantly more often in the intervention group (34 participants [0.16%]) vs the control group (15 participants [0.07%]; *P* = .007). In multivariate analyses involving all 67 683 participants, the use of cimetropium bromide was more likely to detect gastric neoplasms compared with nonuse (odds ratio, 1.42; 95% CI, 1.04-1.95; *P* = .03).

**Conclusions and Relevance:**

In this study, the use of cimetropium bromide as premedication was significantly associated with increased gastric neoplasm detection rates during EGD screening, and lesions in the gastric body were detected more frequently among those who received cimetropium bromide compared with those who did not. These findings suggest that cimetropium bromide may be considered as premedication before EGD examination among individuals with no contraindications.

## Key Points

**Question:**

Is the use of cimetropium bromide, an antispasmodic agent, associated with higher rates of detection of gastric neoplasms during esophagogastroduodenoscopy (EGD) screening?

**Findings:**

In this cohort study of 67 683 participants who received EGD screening, the use of cimetropium bromide as premedication was associated with higher gastric neoplasm detection rates during EGD than nonuse. Lesions in the gastric body were detected more frequently in those who received cimetropium bromide compared with those who did not.

**Meaning:**

This study’s findings suggest that cimetropium bromide may be considered as premedication for the detection of gastric neoplasms during EGD examination among individuals with no contraindications.

## Teaser

This cohort study assesses whether the use of an antispasmodic agent, cimetropium bromide, as premedication is associated with increases in gastric neoplasm detection rates during esophagogastroduodenoscopic examination.

## Introduction

Esophagogastroduodenoscopy (EGD) is a common procedure used to examine upper gastrointestinal (GI) diseases, allowing for direct mucosal visualization and tissue acquisition.^1^ This procedure is also used as a screening tool to identify upper GI cancer in high-risk regions, allowing for detection of early-stage neoplasms, thereby reducing gastric cancer mortality.^2,3^ Because precancerous lesions and early cancers, even some advanced-stage cancers, often show subtle morphological changes on endoscopy, accurate diagnosis requires careful visualization of the mucosa.^4^

During EGD screening, gastric movement interferes with careful examination of the mucosal surface. Therefore, antispasmodic agents have been commonly administered as premedication to inhibit peristalsis during the examination.^5,6,7,8,9^ However, results regarding the benefits of the administration of antispasmodic agents before EGD examination have been inconsistent. Some studies^10,11^ have reported that antispasmodic agents suppress peristalsis and allow for better visualization of mucosal lesions. However, results of other studies^12,13,14^ have not supported these findings. Although there are few data confirming a change in diagnostic yield with the use of antispasmodic agents,^11,15,16^ a recent Asian consensus guideline recommended their use to improve the visual clarity of mucosal inspection before EGD examinations.^17^ In Korea, several antispasmodic agents, such as cimetropium bromide and hyoscine N-butylbromide, have been used as preendoscopic treatment. Cimetropium bromide, a quaternary ammonium compound that is chemically related to scopolamine, competes with acetylcholine to bind to the muscarinic receptors in the GI smooth muscle.^18^ This agent is frequently used to suppress gastric movement. Cimetropium bromide has few serious adverse effects but requires close monitoring when administered to patients with arrhythmia, benign prostatic hypertrophy, and glaucoma.^19,20,21^ The aim of this study was to investigate whether the use of cimetropium bromide as premedication was associated with higher gastric neoplasm detection rates during EGD examinations.

## Methods

We conducted a propensity score–matched retrospective cohort study involving 132 352 participants who received comprehensive health screening at the Health Promotion Center of Seoul St. Mary’s Hospital, The Catholic University of Korea, from January 2, 2010, to June 30, 2017. Data were analyzed from April 1 to December 30, 2021. The study was approved by the institutional review board of Seoul St. Mary’s Hospital. The requirement for informed consent was waived because anonymous data were used. This study followed the principles of the Declaration of Helsinki^22^ and the Strengthening the Reporting of Observational Studies in Epidemiology (STROBE) reporting guideline for cohort studies.

Each participant completed a structured questionnaire that included medical history; family history of cancer; and lifestyle information, such as smoking and alcohol consumption. Participants were included if they were aged 30 to 99 years. We excluded 64 669 participants who had repeat EGD screenings (n = 53 835), experienced GI symptoms that might have been associated with the endoscopic procedure or gastric cancer (n = 9822), received anticholinergic agents within 7 days before the EGD examination (n = 658), had a previous upper GI operation (n = 352), or had incomplete examinations (n = 2). Participant demographic characteristics, endoscopic reports, and histopathological results were retrieved from the hospital’s electronic database.

### Premedication Before EGD Examination

Among all participants, 10 mL of simethicone (Gasocol suspension; Taejoon Pharmaceutical Company) was used as an antifoaming agent. Topical pharyngeal anesthesia was administered using 10% lidocaine spray (Beracaine Spray; Firson Company) among participants without a history of allergy. Sedative endoscopy was performed among those who provided written informed consent for any additional costs, adverse effects, and benefits of sedative medications. For sedation, midazolam (Bukwang Pharmaceutical Company) was injected intravenously. The delivered dose of midazolam was decided by each endoscopist after consideration of the participant’s age and general physical condition.

For the antispasmodic agent, 5 mg of cimetropium bromide (Algiron; Boehringer Ingelheim) was administered intravenously 10 minutes before the EGD examination. In accordance with our institutional policy, all participants were asked about their history of glaucoma, prostatic hyperplasia, or drug allergy, all of which are possible contraindications for the use of anticholinergic agents^6,23^; patients with a history of any of these conditions did not receive cimetropium bromide. Cimetropium bromide was also not administered to patients who did not provide written informed consent for the additional costs, route of administration, and potential adverse events and benefits associated with the medication. No other factors were used to determine use or nonuse of cimetropium bromide, and no other antispasmodic agents were administered to participants who did not receive cimetropium bromide.

### Endoscopic Examination

A total of 14 board-certified endoscopists participated in this study. All endoscopists used the same endoscopy unit (Olympus GIF-Q260 series; Olympus Medical Systems Corporation). Observation time was defined as the time between arrival into the duodenum and withdrawal of the scope. To avoid a possible prolongation of examination time due to biopsy sampling rather than assessment of possible lesions, observation time was assessed only for procedures that did not include biopsies.^24^ To calculate observation time, we used an image archiving system in which endoscopic images showed the time taken. We categorized the endoscopists into 2 groups (fast [≤2:51 minutes] and slow [>2:51 minutes]) using the median value of their observation times. The median observation time for each endoscopist was assessed during the first year after participation in this study. The biopsy rate for each endoscopist was also calculated during the first year after participation in this study and was defined as the proportion of participants with at least 1 biopsy performed during EGD. Endoscopists were again categorized into 2 groups (low [≤18.4%] and high [>18.4%]) based on the median value of their biopsy rates. Atrophic gastritis was evaluated according to the endoscopic atrophic grades described by Kimura and Takemoto.^25^

### Groups and Outcomes

Participants were divided into 2 groups: those who received cimetropium bromide before EGD (intervention group) and those who did not (control group). All gastric lesions were confirmed by histopathological reports. Histopathological diagnosis was established in accordance with the Vienna classification for GI epithelial neoplasia.^26^ The primary outcome was the gastric neoplasm detection rate during EGD screening, with gastric neoplasm detection rate defined as the proportion of study participants with confirmed gastric neoplasms detected during EGD. Gastric neoplasm detection rates excluded the detection of esophageal and duodenal lesions. These types of lesions were excluded because esophageal movement resulting from a patient’s swallowing is not controllable with the use of antispasmodic agents, and the number of duodenal lesions was too small to accurately evaluate the impact of antispasmodic agents. The secondary outcome was the detection rate of small gastric lesions, with a small gastric lesion defined as a neoplasm smaller than 1 cm in diameter. The size of the gastric neoplasm was measured using biopsy forceps.

### Covariates

Regular smoking was defined as smoking more than 1.5 pack-years during the past year. Alcohol drinking was defined as the consumption of more than 14 standard drinks per week for men and more than 7 standard drinks per week for women during the last 6 months.^27^ A standard drink was defined as any type of alcoholic drink that contained more than 12 g of pure alcohol.^28^ *Helicobacter pylori* infection was defined as a positive result on either a urea breath test or an immunoglobin G test for *H pylori*.

### Statistical Analysis

Descriptive statistics for continuous variables were summarized as means with SDs and medians with IQRs. Because all continuous variables did not follow a normal distribution, significant differences between groups were assessed using the nonparametric Wilcoxon rank sum test. Categorical data were described using numbers with percentages and compared using χ^2^ or Fisher exact tests, as appropriate.

For propensity score matching, a 1:1 matching process without replacements was performed using a greedy algorithm with 0.05 caliper (0.25 multiplied by the SD of the logit of the propensity score), yielding 20 835 participants in the intervention group and 20 835 matched participants in the control group. Propensity scores were created using logistic regression modeling of the probability of a patient receiving cimetropium bromide based on age group (31-39 years, 40-59 years, 60-79 years, or ≥80 years), sex (female or male), body mass index (calculated as weight in kilograms divided by height in meters squared; <18.5, 18.5-24.9, 25.0-29.9, or ≥30.0), smoking (yes or no), alcohol drinking (yes or no), family history of gastric cancer (none, first-degree relatives, or other relatives), history of *H pylori* infection (yes or no), grade of atrophic gastritis (none, closed type, or open type), diabetes (yes or no), type of endoscopist by observation time (fast or slow), type of endoscopist by biopsy rate (low or high), and use of midazolam (yes or no). These variables were chosen because they were considered clinically relevant factors that may have been associated with gastric cancer development or neoplasm detection rates during EGD examination.^2,24,29,30,31^ The logistic model including the 12 variables had a C statistic of 0.74. Absolute standardized mean differences were estimated for all baseline covariates before and after matching to assess imbalance before matching and balance after matching. An absolute standardized mean difference of less than 0.1 for a given covariate indicated a relatively small imbalance. We classified unavailable data into a separate *unknown* category for each variable. This category was used to allow the creation of propensity scores without removing variables for which data were missing. Based on propensity score matching, outcomes were compared between groups using the McNemar test, and odds ratios (ORs) were calculated using generalized estimating equations with a log link and an unstructured correction matrix.

We conducted sensitivity analysis to assess whether unknown data produced any bias. Logistic regression analysis was used to identify independent factors associated with the detection of neoplasms in the unmatched cohorts. A multiple logistic regression model was constructed using stepwise selection with entry criteria of *P* ≤ .10 and stay criteria of *P* ≤ .05. Results were reported as ORs with 95% CIs.

For all analyses, 2-tailed *P* < .05 was considered statistically significant. All data were analyzed using SAS software, version 9.4 (SAS Institute Inc).

## Results

### Participant Characteristics

A total of 67 683 participants who received EGD screening were enrolled in the study (Figure). The mean (SD) age was 48.6 (10.8) years; 36 517 participants (54.0%) were male and 31 166 (46.0%) were female (Table 1). All participants were Asian, representing a racially homogenous study population. In total, 28 280 participants (41.8%) received cimetropium bromide before EGD examination, and 39 403 (58.2%) did not. The mean (SD) age was 50.3 (10.6) years in the intervention group and 47.4 (10.8) years in the control group (*P* < .001), and most participants in both groups were male (57.8% in the intervention group and 51.2% in the control group; *P* < .001). Participants in the intervention vs control group had significantly higher body mass index (mean [SD], 23.7 [3.2] vs 23.4 [3.2]; *P* < .001), smoking rates (22.7% vs 15.3%; *P* < .001), alcohol drinking rates (46.5% vs 45.3%; *P* < .001), family history of gastric cancer (first-degree and other relatives: 9.2% vs 7.1%; *P* < .001), history of *H pylori* infection (33.6% vs 20.1%; *P* < .001), atrophic gastritis (closed and open type: 42.0% vs 35.9%; *P* < .001), and diabetes (4.4% vs 2.6%; *P* < .001). The proportion of endoscopists with longer median observation time (ie, >2:51 minutes) was significantly higher among those in the control vs intervention group (44.7% vs 42.1%; *P* < .001). The use of midazolam was also significantly higher in the control vs intervention group (51.9% vs 20.0%; *P* < .001).

**Figure.**
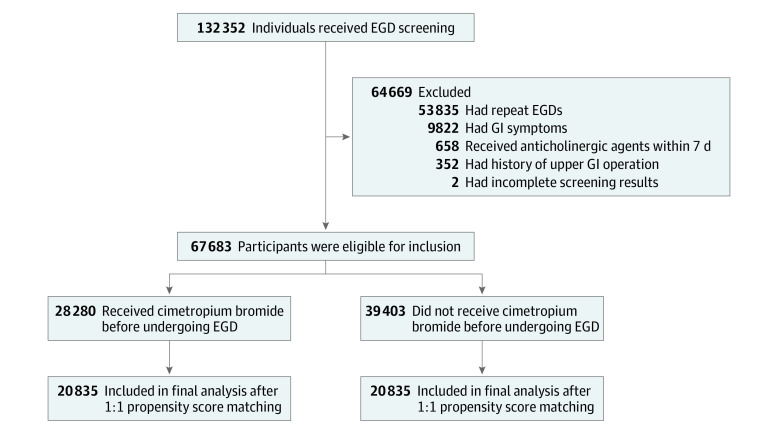
Flow Diagram of Participant Inclusion EGD indicates esophagogastroduodenoscopy; GI, gastrointestinal.

**Table 1.** Participant Characteristics

Characteristic	Unmatched participants					Propensity score–matched (1:1) participants				
	Total, No. (%)	Received cimetropium bromide, No. (%)	Did not receive cimetropium bromide, No. (%)	*P* value^a^	Absolute SMD	Total, No. (%)	Received cimetropium bromide, No. (%)	Did not receive cimetropium bromide, No. (%)	*P* value^a^	Absolute SMD
Total participants, No.	67 683	28 280	39 403	NA	NA	41 670	20 835	20 835	NA	NA
Age, mean (SD), y	48.6 (10.8)	50.3 (10.6)	47.4 (10.8)	<.001	0.26	49.4 (11.1)	49.7 (11.2)	49.0 (11.1)	<.001	0.07
Age group, y										
31-39	15 886 (23.5)	5083 (18.0)	10 803 (27.4)	<.001	0.23	9377 (22.5)	4718 (22.6)	4659 (22.4)	<.001	0.01
40-59	40 806 (60.3)	17 631 (62.3)	23 175 (58.8)		0.07	24 245 (58.2)	11 716 (56.2)	12 529 (60.1)		0.08
60-79	10 729 (15.9)	5488 (19.4)	5241 (13.3)		0.17	7897 (19.0)	4323 (20.7)	3574 (17.2)		0.09
≥80	262 (0.4)	78 (0.3)	184 (0.5)		0.03	151 (0.4)	78 (0.4)	73 (0.4)		0
Sex										
Male	36 517 (54.0)	16 348 (57.8)	20 169 (51.2)	<.001	0.13	24 492 (58.8)	12 073 (57.9)	12 419 (59.6)	<.001	0.03
Female	31 166 (46.0)	11 932 (42.2)	19 234 (48.8)		0.13	17 178 (41.2)	8762 (42.1)	8416 (40.4)		0.03
BMI										
Mean (SD)	23.5 (3.2)	23.7 (3.2)	23.4 (3.2)	<.001	0.09	23.7 (3.2)	23.7 (3.2)	23.7 (3.2)	.49	0
Range										
<18.5	2766 (4.1)	1008 (3.6)	1758 (4.5)	<.001	0.05	1579 (3.8)	805 (3.9)	774 (3.7)	<.001	0.01
18.5-24.9	43 478 (64.2)	17 688 (62.5)	25 790 (65.5)		0.06	26 596 (63.8)	13 335 (64.0)	13 261 (63.6)		0.01
25.0-29.9	18 176 (26.9)	7975 (28.2)	10 201 (25.9)		0.05	11 598 (27.8)	5774 (27.7)	5824 (28.0)		0.01
≥30.0	2166 (3.2)	935 (3.3)	1231 (3.1)		0.01	1416 (3.4)	726 (3.5)	690 (3.3)		0.01
Unknown	1097 (1.6)	674 (2.4)	423 (1.1)		0.10	481 (1.2)	195 (0.9)	286 (1.4)		0.04
Smoking										
No	26 584 (39.3)	11 824 (41.8)	14 760 (37.5)	<.001	0.09	15 201 (36.5)	7625 (36.6)	7576 (36.4)	<.001	0
Yes	12 471 (18.4)	6428 (22.7)	6043 (15.3)		0.19	8157 (19.6)	4275 (20.5)	3882 (18.6)		0.05
Unknown	28 628 (42.3)	10 028 (35.5)	18 600 (47.2)		0.24	18 312 (43.9)	8935 (42.9)	9377 (45.0)		0.04
Alcohol drinking										
No	18 302 (27.0)	7443 (26.3)	10 859 (27.6)	.001	0.03	10 217 (24.5)	5079 (24.4)	5138 (24.7)	.17	0.01
Yes	31 015 (45.8)	13 161 (46.5)	17 854 (45.3)		0.02	18 450 (44.3)	9202 (44.2)	9248 (44.4)		0
Unknown	18 366 (27.1)	7676 (27.1)	10 690 (27.1)		0	13 003 (31.2)	6554 (31.5)	6449 (31.0)		0.01
Family history of gastric cancer										
No	35 986 (53.2)	16 557 (58.5)	19 429 (49.3)	<.001	0.19	21 786 (52.3)	10 872 (52.2)	10 914 (52.4)	<.001	0
First-degree relatives	3491 (5.2)	1717 (6.1)	1774 (4.5)		0.07	2230 (5.4)	1192 (5.7)	1038 (5.0)		0.03
Other relatives	1912 (2.8)	891 (3.2)	1021 (2.6)		0.03	1364 (3.3)	741 (3.6)	623 (3.0)		0.03
Unknown	26 294 (38.8)	9115 (32.2)	17 179 (43.6)		0.24	16 290 (39.1)	8030 (38.5)	8260 (39.6)		0.02
History of *Helicobacter pylori* infection^b^										
No	17 997 (26.6)	9166 (32.4)	8831 (22.4)	<.001	0.23	11 028 (26.5)	5399 (25.9)	5629 (27.0)	<.001	0.03
Yes	17 416 (25.7)	9498 (33.6)	7918 (20.1)		0.31	11 575 (27.8)	6251 (30.0)	5324 (25.6)		0.10
Unknown	32 270 (47.7)	9616 (34.0)	22 654 (57.5)		0.49	19 067 (45.8)	9185 (44.1)	9882 (47.4)		0.07
Atrophic gastritis										
None	41 688 (61.6)	16 413 (58.0)	25 275 (64.1)	<.001	0.13	25 108 (60.3)	12 506 (60.0)	12 602 (60.5)	<.001	0.01
Closed type	21 792 (32.2)	9722 (34.4)	12 070 (30.6)		0.08	13 540 (32.5)	6690 (32.1)	6850 (32.9)		0.02
Open type	4203 (6.2)	2145 (7.6)	2058 (5.2)		0.10	3022 (7.3)	1639 (7.9)	1383 (6.6)		0.05
Diabetes										
No	38 914 (57.5)	17 614 (62.3)	21 300 (54.1)	<.001	0.17	23 200 (55.7)	11 535 (55.4)	11 665 (56.0)	<.001	0.01
Yes	2270 (3.4)	1253 (4.4)	1017 (2.6)		0.10	1717 (4.1)	977 (4.7)	740 (3.6)		0.06
Unknown	26 499 (39.2)	9413 (33.3)	17 086 (43.4)		0.21	16 753 (40.2)	8323 (39.9)	8430 (40.5)		0.01
Type of endoscopist by observation time^c^										
Fast (≤2:51 min)	38 175 (56.4)	16 387 (57.9)	21 788 (55.3)	<.001	0.04	24 050 (57.7)	12 330 (59.2)	11 720 (56.3)	<.001	0.06
Slow (>2:51 min)	29 508 (43.6)	11 893 (42.1)	17 615 (44.7)		0.04	17 620 (42.3)	8505 (40.8)	9115 (43.7)		0.06
Type of endoscopist by biopsy rate^d^										
Low (≤18.4%)	29 454 (43.5)	12 305 (43.5)	17 149 (43.5)	.98	0	18 458 (44.3)	9473 (45.5)	8985 (43.1)	<.001	0.05
High (>18.4%)	38 229 (56.5)	15 975 (56.5)	22 254 (56.5)		0	23 212 (55.7)	11 362 (54.5)	11 850 (56.9)		0.05
Use of midazolam										
No	41 586 (61.4)	22 621 (80.0)	18 965 (48.1)	<.001	0.70	30 577 (73.4)	15 180 (72.9)	15 397 (73.9)	<.001	0.02
Yes	26 097 (38.6)	5659 (20.0)	20 438 (51.9)		0.70	11 093 (26.6)	5655 (27.1)	5438 (26.1)		0.02

Abbreviations: BMI, body mass index (calculated as weight in kilograms divided by height in meters squared); NA, not applicable; SMD, standardized mean difference.

^a^ *P* values were determined using a χ^2^ or Wilcoxon rank sum test for unmatched data and a McNemar or Wilcoxon rank sum test for pair-matched data.

^b^ Positive results for *Helicobacter pylori* infection on either urea breath test or immunoglobulin G test.

^c^ Dichotomized using median value of observation time.

^d^ Dichotomized using median value of biopsy rate.

A total of 41 670 participants (20 835 pairs) who did and did not receive cimetropium bromide were propensity score matched (1:1) based on possible confounding variables. Among matched participants, the mean (SD) age was 49.4 (11.1) years; 24 492 participants (58.8%) were male, and 17 178 (41.2%) were female. Additional baseline characteristics of participants before and after propensity score matching are shown in Table 1.

### Characteristics of Individual Endoscopists

Among 14 endoscopists, 8 were categorized as having fast observation times and 6 as having slow observation times using a median value of 2:51 minutes as the cutoff. The median observation time for a normal EGD examination without biopsy was 2:54 minutes (IQR, 2:24-3:40 minutes) for fast endoscopists and 3:23 minutes (IQR, 2:51-4:12 minutes) for slow endoscopists. A total of 7 endoscopists were categorized as having low biopsy rates and 7 as having high biopsy rates using a median value of 18.4% as the cutoff. The lowest biopsy rate was 188 of 2144 EGD examinations (8.8%), and the highest rate was 693 of 2244 EGD examinations (30.9%). Observation times, biopsy rates, use of midazolam and cimetropium bromide, and detection rate of gastric neoplasms for each endoscopist are shown in Table 2. Additional participant characteristics, including demographic information and smoking and alcohol consumption according to each endoscopist, are available in eTable 1 in the Supplement.

**Table 2.** Baseline Characteristics of Procedures by Individual Endoscopist^a^

Endoscopist	EGD examinations performed, No.	Observation time without biopsy during first year, median (IQR)	No. (%)			
			Biopsy rate	Use of midazolam	Use of cimetropium bromide	Gastric neoplasm
A	2586	2:52 (2:26-3:28)	447 (17.3)	776 (30.0)	1032 (39.9)	3 (0.1)
B	7519	1:47 (1:29-2:12)	1692 (22.5)	2422 (32.2)	3016 (40.1)	17 (0.2)
C	1682	2:37 (2:16-3:07)	424 (25.2)	1093 (65.0)	726 (43.2)	4 (0.2)
D	2283	2:47 (2:27-3:16)	269 (11.8)	780 (34.2)	989 (43.3)	3 (0.1)
E	2244	3:24 (2:43-4:19)	693 (30.9)	659 (29.4)	860 (38.3)	4 (0.2)
F	8928	2:49 (2:28-3:19)	1879 (21.0)	3260 (36.5)	4033 (45.2)	24 (0.3)
G	8990	2:25 (2:05-2:54)	1154 (12.8)	3414 (38.0)	3746 (41.7)	13 (0.1)
H	6371	2:50 (2:28-3:24)	1280 (20.1)	2755 (43.2)	2653 (41.6)	14 (0.2)
I	258	2:36 (2:10-3:07)	23 (8.9)	57 (22.1)	180 (69.8)	1 (0.4)
J	6998	2:59 (2:31-3:35)	1056 (15.1)	3012 (43.0)	2714 (38.8)	18 (0.3)
K	8676	3:37 (3:10-4:11)	2225 (25.6)	3306 (38.1)	3589 (41.4)	32 (0.4)
L	2144	2:51 (2:28-3:22)	188 (8.8)	569 (26.5)	1044 (48.7)	2 (0.1)
M	6195	3:30 (3:02-4:06)	907 (14.6)	2563 (41.4)	2600 (42.0)	16 (0.3)
N	2809	2:56 (2:36-3:19)	548 (19.5)	1431 (50.9)	1098 (39.1)	9 (0.3)
Type of endoscopist by observation time^b^						
Fast (≤2:51 min)	38 175	2:54 (2:24-3:40)	6909 (18.1)	14 350 (37.6)	16 387 (42.9)	78 (0.2)
Slow (>2:51 min)	29 508	3:23 (2:51-4:12)	5876 (19.9)	11 747 (39.8)	11 893 (40.3)	82 (0.3)

Abbreviation: EGD, esophagogastroduodenoscopy.

^a^ *P* values were <.001 for all comparisons with the exception of gastric neoplasm, for which the *P* value was .05. *P* values were calculated using a Wilcoxon rank sum test or χ^2^ test.

^b^ Dichotomized using median value of observation time. A total of 8 endoscopists were included in the fast category, and 6 endoscopists were included in the slow category.

### Gastric Neoplasms Detected During EGDs

Among 41 670 participants in the propensity score–matched cohorts, gastric neoplasms were diagnosed in 102 individuals (0.24%) (Table 3). A total of 52 dysplasias (0.12%), 40 early cancers (0.10%), 7 advanced cancers (0.02%), and 3 lymphomas (0.01%) were detected. In the propensity score–matched cohorts, small gastric lesions (<1 cm) comprised 35 of the total 102 gastric neoplasms (34.3%), and most neoplasms were found in the gastric body (49 participants [48.0%]) and antrum (49 participants [48.0%]).

**Table 3.** Diagnostic Outcomes of Unmatched and Propensity Score–Matched Groups

Outcome	No. (%)							
	Unmatched participants				Propensity score–matched (1:1) participants			
	Total (n = 67 683), No. (%)	Received cimetropium bromide (n = 28 280), No. (%)	Did not receive cimetropium bromide (n = 39 403), No. (%)	*P* value^a^	Total (n = 41 670), No. (%)	Received cimetropium bromide (n = 20 835), No. (%)	Did not receive cimetropium bromide (n = 20 835), No. (%)	*P* value^a^
Total gastric neoplasms	160 (0.24)	87 (0.31)	73 (0.19)	.001	102 (0.24)	63 (0.30)	39 (0.19)	.02
Dysplasia	85 (0.13)	44 (0.16)	41 (0.10)	.06	52 (0.12)	32 (0.15)	20 (0.10)	.10
Early cancer	61 (0.09)	36 (0.13)	25 (0.06)	.006	40 (0.10)	25 (0.12)	15 (0.07)	.11
Advanced cancer	10 (0.01)	6 (0.02)	4 (0.01)	.34	7 (0.02)	5 (0.02)	2 (0.01)	.26
Lymphoma	3 (0.004)	1 (0.004)	2 (0.01)	>.99	3 (0.01)	1 (0.005)	2 (0.01)	.56
Neuroendocrine tumor	1 (0.001)	0	1 (0.003)	>.99	0	0	0	NA
Dysplasia and early cancer	146 (0.22)	80 (0.28)	66 (0.17)	.002	92 (0.22)	57 (0.27)	35 (0.17)	.02
Small gastric neoplasms^b^	56 (0.08)	35 (0.12)	21 (0.05)	.002	35 (0.08)	24 (0.12)	11 (0.05)	.03
Dysplasia	42 (0.06)	26 (0.09)	16 (0.04)	.008	26 (0.06)	18 (0.09)	8 (0.04)	.05
Early cancer	14 (0.02)	9 (0.03)	5 (0.01)	.09	9 (0.02)	6 (0.03)	3 (0.01)	.32
Location								
Cardia and fundus	5 (0.01)	2 (0.01)	3 (0.01)	>.99	4 (0.01)	2 (0.01)	2 (0.01)	>.99
Body	72 (0.11)	44 (0.16)	28 (0.07)	.001	49 (0.12)	34 (0.16)	15 (0.07)	.007
Antrum	82 (0.12)	40 (0.14)	42 (0.11)	.20	49 (0.12)	27 (0.13)	22 (0.11)	.48

Abbreviation: NA, not available.

^a^ *P* values were determined using an χ^2^ test or a Fisher exact test for unmatched data and a McNemar test for matched data.

^b^ Neoplasms <1 cm in diameter.

### Gastric Neoplasm Detection Rates

In the propensity score–matched cohorts, neoplasm detection rates were significantly higher in the intervention group (63 participants [0.30%]) than the control group (39 participants [0.19%]; *P* = .02) (Table 3). When only accounting for dysplasia and early gastric cancer, which are potentially indicative of the need for future endoscopic resection, the combined detection rate remained significantly higher in the intervention group (57 participants [0.27%]) vs the control group (35 participants [0.17%]; *P* = .02). The detection rate of small gastric lesions was also significantly higher in the intervention group (24 participants [0.12%]) vs the control group (11 participants [0.05%]; *P* = .03), and neoplasms in the gastric body were more frequently found in the intervention group (34 participants [0.16%]) vs the control group (15 participants [ 0.07%]; *P* = .007) (Table 3). A sensitivity analysis using multiple imputations was performed to assess whether unknown data produced any bias. The use of cimetropium bromide was consistently associated with higher gastric neoplasm detection rates (pooled OR, 1.55; 95% CI, 1.03-2.33; *P* = .04) (eTable 2 in the Supplement).

### Factors Associated With Gastric Neoplasm Detection Rates

In the univariate analysis of unmatched data, older age (≥80 years: OR, 62.63; 95% CI, 25.24-155.40; *P* < .001), male sex (OR, 2.11; 95% CI, 1.50-2.96; *P* < .001), smoking (OR, 1.55; 95% CI, 1.03-2.33; *P* = .04), history of *H pylori* infection (OR, 4.01; 95% CI, 2.44-6.61; *P* < .001), atrophic gastritis (OR, 4.39; 95% CI, 2.85-6.76; *P* < .001), high biopsy rate (OR, 1.43; 95% CI, 1.03-1.97; *P* = .03), and use of cimetropium bromide (OR, 1.66; 95% CI, 1.22-2.27; *P* = .001) were significantly associated with higher gastric neoplasm detection rates (Table 4). The multivariate analysis revealed that the use of cimetropium bromide was more likely to detect gastric neoplasms compared with nonuse (OR, 1.42; 95% CI, 1.04-1.95; *P* = .03) after adjusting for older age, male sex, low body mass index, *H pylori* infection, atrophic gastritis, endoscopists with slow observation times, and endoscopists with high biopsy rates. Even after including all variables, the multivariate analysis revealed a consistent association between the use of cimetropium bromide and higher gastric neoplasm detection rates (OR, 1.54; 95% CI, 1.11-2.13; *P* = .009) (eTable 3 in the Supplement).

**Table 4.** Univariate and Multivariate Logistic Regression Analyses of Factors Associated With Detection of Gastric Neoplasm

Variable	No. (%)		Univariate analysis		Multivariate analysis^a^	
	Gastric neoplasm (n = 160)	Nongastric neoplasm (n = 67 523)	OR (95% CI)	*P* value	Adjusted OR (95% CI)	*P* value
Age group, y						
31-39	9 (5.6)	15 877 (23.5)	1 [Reference]	NA	1 [Reference]	NA
40-59	71 (44.4)	40 735 (60.3)	2.93 (1.49-5.77)	.002	1.80 (0.91-3.56)	.09
60-79	71 (44.4)	10 658 (15.8)	11.21 (5.70-22.07)	<.001	4.37 (2.14-8.91)	<.001
≥80	9 (5.6)	253 (0.4)	62.63 (25.24-155.40)	<.001	18.70 (7.15-48.90)	<.001
Sex						
Male	114 (71.3)	36 403 (53.9)	2.11 (1.50-2.96)	<.001	2.04 (1.45-2.88)	<.001
Female	46 (28.8)	31 120 (46.1)	1 [Reference]		1 [Reference]	
BMI range						
<18.5	11 (6.9)	2755 (4.1)	1.78 (0.97-3.29)	.06	2.74 (1.47-5.08)	.001
18.5-24.9	101 (63.1)	43 377 (64.2)	1 [Reference]	NA	1 [Reference]	NA
25.0-29.9	38 (23.8)	18 138 (26.9)	0.91 (0.63-1.32)	.61	0.73 (0.50-1.06)	.10
≥30.0	4 (2.5)	2162 (3.2)	0.89 (0.35-2.29)	.81	0.98 (0.39-2.50)	.97
Unknown	6 (3.8)	1091 (1.6)	2.55 (1.15-5.64)	.02	1.67 (0.73-3.80)	.22
Smoking						
No	54 (33.8)	26 530 (39.3)	1 [Reference]	NA	[Reference]	NA
Yes	39 (24.4)	12 432 (18.4)	1.55 (1.03-2.33)	.04	NA	NA
Unknown	67 (41.9)	28 561 (42.3)	1.15 (0.80-1.64)	.44	NA	NA
Alcohol drinking						
No	48 (30.0)	18 254 (27.0)	1 [Reference]	NA	1 [Reference]	NA
Yes	67 (41.9)	30 948 (45.8)	0.82 (0.57-1.19)	.30	NA	NA
Unknown	45 (28.1)	18 321 (27.1)	0.93 (0.62-1.40)	.74	NA	NA
Family history of gastric cancer						
No	84 (52.5)	35 902 (53.2)	1 [Reference]	NA	1 [Reference]	NA
First-degree relatives	10 (6.3)	3481 (5.2)	1.28 (0.67-2.44)	.45	NA	NA
Other relatives	4 (2.5)	1908 (2.8)	1.00 (0.39-2.59)	>.99	NA	NA
Unknown	62 (38.8)	26 232 (38.8)	1.01 (0.73-1.40)	.94	NA	NA
History of *Helicobacter pylori* infection						
No	19 (11.9)	17 978 (26.6)	1 [Reference]	NA	1 [Reference]	NA
Yes	75 (46.9)	17 341 (25.7)	4.01 (2.44-6.61)	<.001	2.11 (1.27-3.49)	.004
Unknown	66 (41.3)	32 204 (47.7)	1.90 (1.15-3.15)	.01	2.00 (1.20-3.35)	.008
Atrophic gastritis						
No	29 (18.1)	41 659 (61.7)	1 [Reference]	NA	1 [Reference]	NA
Yes	67 (41.9)	21 725 (32.2)	4.39 (2.85-6.76)	<.001	2.91 (1.87-4.55)	<.001
Unknown	64 (40.0)	4139 (6.1)	22.00 (14.22-34.06)	<.001	9.73 (6.03-15.70)	<.001
Diabetes						
No	94 (58.8)	38 820 (57.5)	1 [Reference]	NA	1 [Reference]	NA
Yes	7 (4.4)	2263 (3.4)	1.36 (0.65-2.87)	.42	NA	NA
Unknown	59 (36.9)	26 440 (39.2)	0.92 (0.67-1.28)	.64	NA	NA
Type of endoscopist by observation time^b^						
Fast (≤2:51 min)	78 (48.8)	38 097 (56.4)	1 [Reference]	.05	1 [Reference]	.01
Slow (>2:51 min)	82 (51.3)	29 426 (43.6)	1.36 (1.00-1.85)		1.49 (1.09-2.04)	
Type of endoscopist by biopsy rate^c^						
Low (≤18.4%)	56 (35.0)	29 398 (43.5)	1 [Reference]	.03	1 [Reference]	.007
High (>18.4%)	104 (65.0)	38 125 (56.5)	1.43 (1.03-1.97)		1.56 (1.13-2.15)	
Use of midazolam						
No	102 (63.8)	41 484 (61.4)	1 [Reference]	.56	1 [Reference]	NA
Yes	58 (36.3)	26 039 (38.6)	0.91 (0.66-1.25)		1.31 (0.94-1.83)	
Use of cimetropium bromide						
No	73 (45.6)	39 330 (58.2)	1 [Reference]	.001	1 [Reference]	.03
Yes	87 (54.4)	28 193 (41.8)	1.66 (1.22-2.27)		1.42 (1.04-1.95)	

Abbreviations: BMI, body mass index (calculated as weight in kilograms divided by height in meters squared); NA, not applicable; OR, odds ratio.

^a^ Stepwise selection with entry criteria of *P* ≤ .10 and stay criteria of *P* ≤ .05.

^b^ Dichotomized using median value of observation time.

^c^ Dichotomized using median value of biopsy rate.

## Discussion

This cohort study found that the use of an antispasmodic agent, cimetropium bromide, as premedication was associated with higher gastric neoplasm detection rates during EGD examinations. We also found that the use of cimetropium bromide before EGD screening was more likely to increase the detection rates of gastric dysplasia and early cancer, which are the ultimate targets of endoscopic screening. Furthermore, the detection rate of small gastric neoplasms was significantly higher among those who received cimetropium bromide before EGD screening than those who did not.

Mucosal visualization during EGD examination is often limited by spasm or peristalsis of the GI tract, which hinders detailed observation and may result in missed detection of subtle gastric neoplasms.^4,32^ Improved visualization through the use of antispasmodic agents could theoretically enhance the detection of lesions during EGD examination. However, there is considerable variation in the routine use of antispasmodic agents based on the experiences and preferences of each country, institution, or endoscopist.^33^ In addition, there is no consensus on the use of antispasmodic agents.^34,35,36^

A recent Asian consensus guideline^17^ recommended the routine use of antispasmodic agents before EGD examination to increase the detection rate of precancerous neoplasms, even when there is a paucity of evidence to support this recommendation. However, a previous study^37^ reported that the use of scopolamine did not significantly increase the detection of upper GI neoplasms. The study enrolled 6625 pairs of participants who did and did not receive scopolamine before EGD examination, with propensity score matching performed based on possible covariates that may have been associated with neoplasm detection rates. A total of 54 lesions were found in the esophagus, stomach, and duodenum. The researchers concluded that the use of scopolamine was not associated with improvements in the detection rate of any upper GI neoplasm.^37^

Compared with the previous study,^37^ our study conducted an analysis among a sample that was 3 times larger. We focused on gastric neoplasm detection rates after excluding esophageal and duodenal lesions because esophageal movement cannot be controlled by antispasmodic agents, and the number of duodenal lesions was too small to accurately evaluate the impact of cimetropium bromide. Furthermore, we included other important confounders for gastric neoplasm detection rates, such as *H pylori* infection, grade of atrophic gastritis, and endoscopist observation time.^24,38^

Several antispasmodic agents, such as hyoscine butylbromide, glucagon, and cimetropium bromide, have been used clinically as premedication before GI endoscopic evaluations and have had good safety profiles. The Korea Institute of Drug Safety and Risk Management–Korea Adverse Event Reporting System reports cases of anaphylaxis and death associated with medication use. According to a study of case safety reports^39^ recorded in this system between January 2008 and December 2017, 4700 cases of drug-induced anaphylaxis and 8664 deaths associated with drugs were reported. However, there were no descriptions of anaphylaxis or death associated with antispasmodic agents, including cimetropium bromide. Consistent with this finding, no serious adverse events were reported in our study.

The present study found that cimetropium bromide was useful for detecting neoplasms located in the gastric body. During colonoscopy, antispasmodic agents also assist in flattening haustral folds and enhance visualization of the mucosa behind the folds to improve the detection rates of colon polyps and dysplasia.^40^ Because the presence of multiple folds in the gastric body presents obstacles during observation that are similar to those of haustral folds in the colon, the impact of antispasmodic agents may also be substantial. When peristalsis is increased by the stimulation of scope insertion during EGD screening, neoplasms located in the gastric body may not be easily detected in a contracted or incompletely dilated stomach. A previous systematic review^41^ reported that missed gastric cancers were mainly located in the gastric body. Therefore, the use of antispasmodic agents may reduce the contraction of the gastric body and decrease the rates of missed detection of gastric neoplasms. In the present study, the detection rates of neoplasms in the antrum were not significantly different between the 2 groups. This finding might be explained by the fact that the antrum is more easily exposed, even in cases of increased peristalsis, compared with other locations.^42^

### Strengths and Limitations

This study has several strengths. First, we adjusted for many confounding factors that may have been associated with gastric neoplasm detection rates, including participant characteristics and endoscopist factors, such as biopsy rate and observation time. Second, we minimized the impact of endoscopists’ experience by including only board-certified endoscopists, thereby reducing variations associated with endoscopists’ differing levels of expertise. Third, we included more than 60 000 participants to provide adequate numbers for the analysis. Previous studies^24,43^ reported that the incidence of gastric neoplasms was less than 0.3% among participants who received screening. Therefore, a large number of participants were required to accurately assess the association of an antispasmodic agent with gastric neoplasm detection rates during EGD screening. Fourth, we analyzed the detection rate of small lesions as well as total gastric neoplasms to provide reliable results regarding the impact of cimetropium bromide, with both analyses yielding consistent findings.

The study also has limitations. First, the cohort included participants with unavailable data for some covariates, which might be a source of bias in a retrospective study. We dealt with these unavailable data by adding an *unknown* category for each variable. This method required approximation that may have produced variance in the data. Second, cimetropium bromide may not be available in all countries. However, it is worth noting that this drug has antiperistaltic benefits that are almost equivalent to those of hyoscine N-butylbromide, a widely used antispasmodic agent.^8,9,10,23^ Third, we could not fully investigate minor adverse events because no additional medical reports of minor events were recorded by our institution. Fourth, we could not directly evaluate the extent of peristalsis after the injection of cimetropium bromide during EGD examination. Different individuals may have different responses to cimetropium bromide. However, the measurement of peristalsis is difficult and not validated. Further research is needed to identify objective methods for combining the evaluations of antiperistaltic activity and gastric neoplasm detection rates.

## Conclusions

This cohort study found a significant association between the use of cimetropium bromide as premedication and improvements in the detection rate of total gastric neoplasms, gastric dysplasia, and early gastric cancer during EGD screening. These findings suggest that cimetropium bromide may be considered for use as premedication before EGD examination among individuals with no contraindications.

## Notes

Supplement. **eTable 1.** Baseline Characteristics of Participants According to Endoscopists **eTable 2.** Odds Ratios From Propensity Score Matching After Multiple Imputation **eTable 3.** Univariate and Multivariate Logistic Regression Analyses for Factors Associated With Detection of Gastric Neoplasm
