# Supplementary material for: Assessment of Cimetropium Bromide Use for the Detection of Gastric Neoplasms During Esophagogastroduodenoscopy
Source: JAMA Netw Open. 2022 Mar 23;5(3):e223827. doi: 10.1001/jamanetworkopen.2022.3827 (PMC8943631; doi:10.1001/jamanetworkopen.2022.3827)
Supplement: Supplement. — eTable 1. Baseline Characteristics of Participants According to Endoscopists eTable 2. Odds Ratios From Propensity Score Matching After Multiple Imputation eTable 3. Univariate and Multivariate Logistic Regression Analyses for Factors Associated With Detection of Gastric Neoplasm [file jamanetwopen-e223827-s001.pdf]

Supplementary Online Content

Kim SY, Park JM, Cho HS, Cho YK, Choi MG. Assessment of cimetropium bromide use for the detection of gastric neoplasms during esophagogastroduodenoscopy. *JAMA Netw Open*. 2022;5(3):e223827. doi:10.1001/jamanetworkopen.2022.3827

eTable 1. Baseline Characteristics of Participants According to Endoscopists

eTable 2. Odds Ratios From Propensity Score Matching After Multiple Imputation

eTable 3. Univariate and Multivariate Logistic Regression Analyses for Factors Associated With Detection of Gastric Neoplasm

This supplementary material has been provided by the authors to give readers additional information about their work.

© 2022 Kim SY et al. *JAMA Network Open*.

eTable 1. Baseline Characteristics of Participants According to Endoscopists

Endoscopist	Age, Mean ± SD	Men, n (%)	Female, n (%)	BMI, N; Mean ± SD	Smoking, n (%)	Alcohol drinking, n (%)
A	48.9 ± 10.3	1456 (56.3)	1130 (43.7)	2556; 23.6 ± 3.1	477 (18.5)	929 (35.9)
B	48.5 ± 10.4	4009 (53.3)	3510 (46.7)	7398; 23.6 ± 3.2	1426 (19.0)	3788 (50.4)
C	47.2 ± 10.9	945 (56.2)	737 (43.8)	1674; 23.5 ± 3.3	256 (15.2)	427 (25.4)
D	48.9 ± 10.8	1253 (54.9)	1030 (45.1)	2230; 23.6 ± 3.3	333 (14.6)	1389 (60.8)
E	49.0 ± 10.0	1237 (55.1)	1007 (44.9)	2206; 23.6 ± 3.1	427 (19.0)	791 (35.3)
F	49.8 ± 11.4	5009 (56.1)	3919 (43.9)	8774; 23.6 ± 3.2	1955 (21.9)	4212 (47.2)
G	48.3 ± 10.7	4800 (53.4)	4190 (46.6)	8863; 23.5 ± 3.2	1664 (18.5)	4111 (45.7)
H	48.2 ± 10.9	3420 (53.7)	2951 (46.3)	6271; 23.5 ± 3.2	1184 (18.6)	3213 (50.4)
I	51.8 ± 11.2	124 (48.1)	134 (51.9)	258; 23.8 ± 2.8	0 (0.0)	0(0.0)
J	48.3 ± 10.7	3723 (53.2)	3275 (46.8)	6864; 23.5 ± 3.2	1299 (18.6)	3457 (49.4)
K	48.4 ± 10.9	4695 (54.1)	3981 (45.9)	8523; 23.6 ± 3.3	1639 (18.9)	4048 (46.7)
L	50.9 ± 10.7	1207 (56.3)	937 (43.7)	2111; 23.6 ± 3.2	336 (15.7)	611 (28.5)
M	48.1 ± 10.6	3189 (51.5)	3006 (48.5)	6084; 23.4 ± 3.3	1075 (17.4)	2943 (47.5)
N	47.5 ± 11.0	1450 (51.6)	1359 (48.4)	2774; 23.5 ± 3.3	400 (14.2)	1096 (39.0)
Types of endoscopist (observation time)[a]						
Fast (≤ 2:51 min, n=8)	49.2 ± 1.5	18 797 (54.3)	17 408 (45.6)	23.6 ± 0.1	7154 (18.7)	17 751 (46.5)
Slow (>2:51 min, n=6)	48.4 ±	17 720	13	23.5 ± 0.1	5317	13 264

© 2022 Kim SY et al. *JAMA Network Open*.

	0.6	(53.6)	758 (46.6)		(18.0)	(45.0)
P	.439	.008		.519	< .001	< .001

P values were calculated using Wilcoxon rank sum test or chi-square.
Abbreviations: BMI, body mass index; SD, standard deviation.
[a] dichotomized using median value.

© 2022 Kim SY et al. *JAMA Network Open*.

eTable 2. Odds Ratios From Propensity Score Matching After Multiple Imputation

We conducted the multiple imputations to account for missing values for BMI, smoking, alcohol drinking, family history of gastric cancer, *H. pylori* infection, and diabetes mellitus. Multiple imputation using fully conditional specification (FCS) was used to replace missing data and imputation model included all predict variables and total gastric neoplasms. We generated 50 imputation data sets and number of imputations were sufficient. [Fraction Missing Information (FMI) was 0.16, and ≥ 100 x FMI]. Rubin's rule was used to combine the multiple imputed estimates.

	Cimetropium bromide		OR (95% CI)	*P value*
	Users	Non-users		
Total gastric neoplasms				
PSM (n=47 174) -1 imputed dataset	70 (0.30)	43 (0.18)	1.63 (1.11-2.38)	.012
PSM (n=47 128) -10 imputed dataset	74 (0.31)	40 (0.17)	1.85 (1.26-2.71)	.002
PSM (n=47 278) -20 imputed dataset	75 (0.32)	44 (0.19)	1.71 (1.18-2.48)	.005
PSM (n=47 270) -30 imputed dataset	73 (0.31)	43 (0.18)	1.70 (1.17-2.47)	.005
PSM (n=47 220) -40 imputed dataset	75 (0.32)	43 (0.18)	1.75 (1.20-2.54)	.004
PSM (n=47 232) -50 imputed dataset	72 (0.30)	46 (0.19)	1.57 (1.08-2.26)	.017
Pooled			1.55 (1.03-2.33)	.036
Abbreviations: OR, odds ratio; CI, confidence interval; PSM, propensity score-matching.				

© 2022 Kim SY et al. *JAMA Network Open*.

eTable 3. Univariate and Multivariate Logistic Regression Analyses for Factors Associated With Detection of Gastric Neoplasm

	Univariate		Multivariate[c]	
	OR (95% CI)	*P* value	Adjust OR (95% CI)	*P* value
Age, year				
31-39	1		1	
40-59	2.93 (1.49-5.77)	.002	1.88 (0.96-3.69)	.068
60-79	11.21 (5.70-22.07)	< .001	4.78 (2.35-9.70)	< .001
≥80	62.63 (25.24-155.4)	< .001	22.27 (8.44-58.75)	< .001
Sex				
Men	2.11 (1.50-2.96)	< .001	2.07 (1.41-3.03)	< .001
Women	1		1	
Body mass index (kg/m^2)				
<18.5	1.78 (0.97-3.29)	.064	2.69 (1.46-4.97)	.002
18.5–24.9	1		1	
25–29.9	0.91 (0.63-1.32)	.607	0.74 (0.51-1.07)	.108
≥30	0.89 (0.35-2.29)	.808	0.99 (0.39-2.49)	.985
Unknown	2.55 (1.15-5.64)	.021	1.76 (0.75-4.16)	.195
Smoking				
No	1		1	
Yes	1.55 (1.03-2.33)	.037	1.42 (0.92-2.20)	.114
Unknown	1.15 (0.80-1.64)	.442	1.61 (0.68-3.81)	.281
Alcohol drinking				
No	1		1	
Yes	0.82 (0.57-1.19)	.295	0.80 (0.53-1.19)	.261
Unknown	0.93 (0.62-1.40)	.744	0.70 (0.43-1.15)	.156
Family history of gastric cancer				
No	1		1	
First degree	1.28 (0.67-2.44)	.449	1.08 (0.57-2.02)	.816
Other relatives	1.00 (0.39-2.59)	.997	1.41 (0.56-3.58)	.469
Unknown	1.01 (0.73-1.40)	.942	1.78 (0.74-4.30)	.198
H. pylori infection				
No	1		1	
Yes	4.01 (2.44-6.61)	< .001	2.12 (1.29-3.49)	.003
Unknown	1.90 (1.15-3.15)	.013	1.72 (1.01-2.93)	.045
Grade of atrophic gastritis				
No	1		1	

© 2022 Kim SY et al. *JAMA Network Open*.

Yes	4.39 (2.85-6.76)	< .001	2.90 (1.86-4.50)	< .001
Unknown	22.00 (14.22-34.06)	< .001	9.68 (6.03-15.54)	< .001
Diabetes mellitus				
No	1		1	
Yes	1.36 (0.65-2.87)	.417	0.56 (0.27-1.16)	.12
Unknown	0.92 (0.67-1.28)	.635	0.52 (0.25-1.10)	.088
Type of endoscopist (observation time)[a]				
Fast (≤2:51 min, median)	1		1	
Slow (>2:51 min, median)	1.36 (1.00–1.85)	.051	1.51 (1.11-2.06)	.009
Type of endoscopist (biopsy rate)[b]				
Low (≤18.4%, median)	1		1	
High (>18.4%, median)	1.43 (1.03-1.97)	.032	1.59 (1.15-2.19)	.005
Use of midazolam				
No	1		1	
Yes	0.91 (0.66-1.25)	.562	1.31 (0.94-1.83)	.116
Use of cimetropium bromide				
No	1		1	
Yes	1.66 (1.22-2.27)	.001	1.54 (1.11-2.13)	.009

Abbreviations: OR, odds ratio; CI, confidence interval.
[a] Dichotomized using median value of observation time.
[b] Dichotomized using median value of biopsy rate.
[c] All variables were included

© 2022 Kim SY et al. *JAMA Network Open*.
